# 1850. Purpura Fulminans and Invasive Streptococcus pneumoniae in Immunocompetent individual

**DOI:** 10.1093/ofid/ofac492.1479

**Published:** 2022-12-15

**Authors:** Talha Perwez, Nabil Zeineddine, Junaid Farooq, F N U Duremala, Abrar Khan

**Affiliations:** SUNY Upstate Medical University, Syracuse, New York; SUNY Upstate Medical University, Syracuse, New York; SUNY Upstate Medical University, Syracuse, New York; University of Alabama, Montgomery, Montgomery, Alabama; SUNY Upstate Medical University, Syracuse, New York

## Abstract

**Background:**

Purpura Fulminans (PF) associated with invasive Streptococcus pneumoniae (ISP) is extremely rare. We present a case of PF associated with ISP in an immunocompetent person.

**Methods:**

45-year-old male previously healthy, presented with fall, and dizziness for 1 day, preceded by bloody bowel movements and abdominal pain. On arrival to ED, he had shortness of breath, chest tightness and headache. He had a temperature of 101.7 F, HR 128 BPM, RR 37 BPM, and SpO2 94% RA. He had coarse breath sounds bilaterally, petechial and purpuric rash on chest, lips, bilateral upper and lower extremities. Laboratory findings were significant for WBC 39.3 10*3/uL, Hemoglobin 9.3 g/dL, platelets 33 10*3/uL, creatinine 2.8 mg/dl, AST 523, ALT 242, total bilirubin 3.2 with direct 1.5. INR was 2.86, PT 28.9, PTT 57.2, D dimer >20, and fibrinogen 175. CK was 16,295, ferritin 5294, and LDH 1749. Blood smear revealed schistocytes. Blood cultures grew Streptococcus pneumoniae. He was found to be in DIC, with multi organ failure. Over the course of stay he required O2 support with HFNC of 30 L/min. CT chest showed bilateral ground glass opacities. CT A/P was unremarkable. Autoimmune work up including ANA, ANCA, completement and immunoglobulin levels were unremarkable. HIV and hepatitis panel were negative. TTE negative for vegetations. LP could not be performed due to low platelets. He was started on ceftriaxone 2gm IV Q12. He was given 2 doses of IVIG and 3 days of methylprednisolone 1gm due to initial concerns IgA vasculitis.

**Figure:**
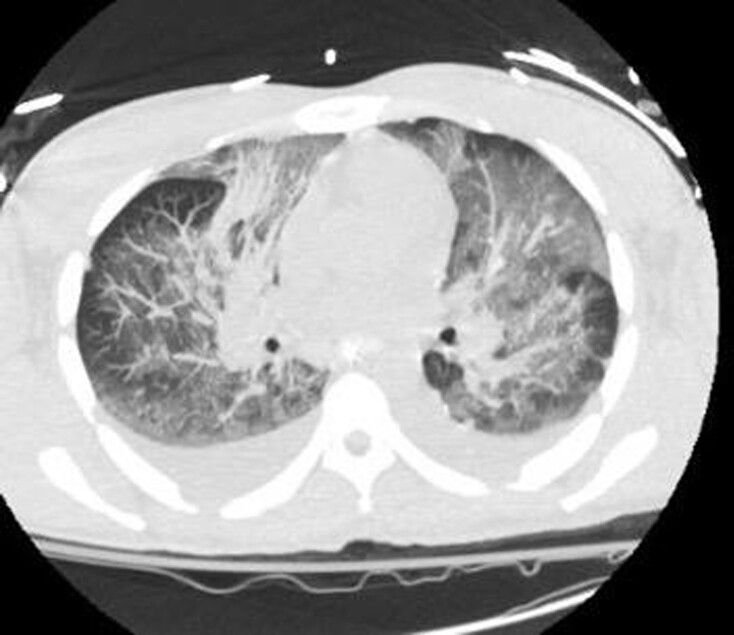

**Results:**

However, skin biopsy revealed small vessel neutrophilic vasculitis with thromboses consistent with purpura fulminan.

**Figure:**
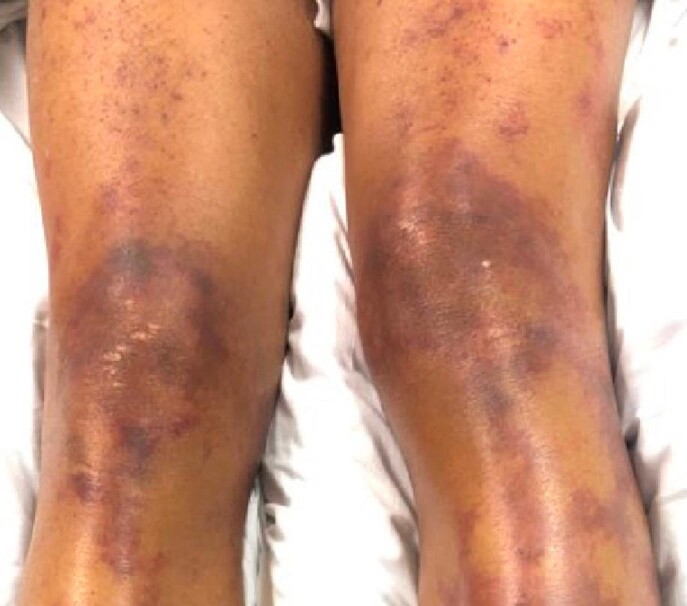

**Figure:**
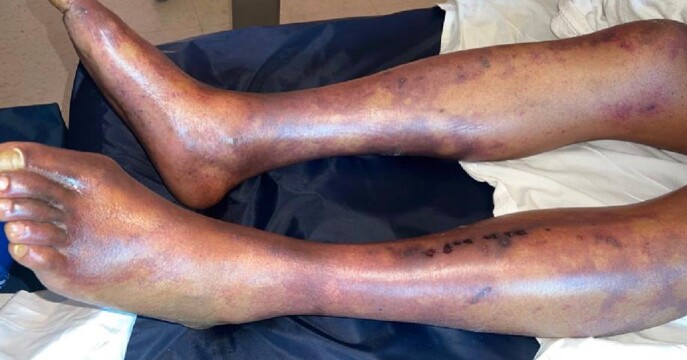

**Figure:**
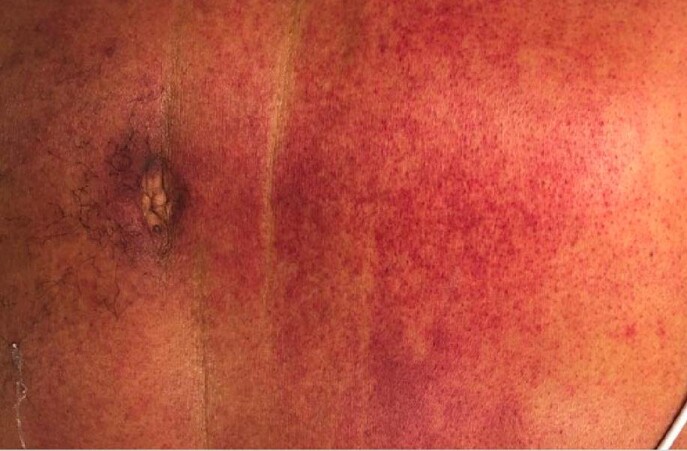

**Conclusion:**

PF is an acute purpuric rash characterized by coagulation of the microvasculature, leading to purpuric lesions and skin necrosis. It is rapidly progressive and is often accompanied by disseminated intravascular coagulation and circulatory collapse. However, it is extremely rare in immunocompetent individuals. Upon our literature review there are only handful of case reports that has described the association of ISP with PF in immunocompetent individual. Our case highlights such rare association and stress on early recognition and treatment as it can cause significant morbidity and mortality.

**Disclosures:**

**All Authors**: No reported disclosures.

